# Utilization of emergency medical service and its associated factors among patients visited public hospitals at Hawassa City, Sidama Region, Ethiopia, 2023

**DOI:** 10.1016/j.heliyon.2024.e31906

**Published:** 2024-05-23

**Authors:** Zelalem Mekonen, Wegene Jemebere, Aklile Tsega Chekol, Fikru Tadesse, Yacob Abraham Borie, Ezedin Mola, Mastewal Aschale Wale, Yunuka Marufa Tunushe, Yared Reta, Amdehiwot Aynalem, Beyene Feleke, Gelane Geleto Gobena, Bereket Beyene, Tomas Yeheyis

**Affiliations:** a Adare General Hospital, Hawassa, Ethiopia; b School of Nursing, College of Medicine and Health Sciences, Hawassa University, Ethiopia; c Hawassa University Comprehensive Specialized Hospital, Hawassa, Ethiopia; d School of Nursing Haramaya University, Haramaya, Ethiopia

**Keywords:** Emergency medical service, Pre-hospital care, Associated factors, Utilization, Hawassa, Ethiopia

## Abstract

**Background:**

The burden of emergency medical conditions is borne mostly by poorer nations, with a 6 % increase in deaths of adults and children due to emergency conditions between 1990 and 2015. Emergency medical service is crucial to improve outcomes of those injuries and other time-sensitive illnesses. However, access to emergency medical services in Hawassa City is still limited and its’ utilization is influenced by different factors.

**Methods:**

A facility-based cross-sectional study was conducted among 422 randomly selected clients who visited the emergency service in public hospitals of Hawassa City. A structured interviewer-administered questionnaire adapted by reviewing previous literature was used. The collected data by using the Kobo toolbox was exported into a statical package for social science software for analysis. Descriptive statistics such as frequency, percentage, mean, and standard deviation were used. A binary logistic regression model at a 95 % confidence interval was used to declare an association between dependent and independent variables using the odds ratio.

**Results:**

All 422 participants completed the interview with a response rate of 100 %. The mean age of the study participants was 33.73 years with a 14.67 standard deviation. One quarter (24.9 % (95 % CI: 21.1–29.4)) of the study participants have utilized emergency medical services. Urban residence (AOR = 3.48, 95 % CI: 1.69–7.16), ever utilized ambulance service (AOR = 2.37, 95%CI: 1.21–4.67), having Red Cross Association ambulance number (AOR = 2.64, 95%CI: 1.20–5.83) and awareness on presence of free government ambulance (AOR = 3.74, 95%CI: 1.46–9.59) were the predictors of the outcome variable.

**Conclusion:**

utilization of emergency medical services in the study area was relatively low when compared with other studies. urban residence, ever utilization of ambulance service, awareness of the presence of free government ambulances, and having a Red Cross Association ambulance number were predictors of utilization of emergency medical service.

## 1 Introduction

The burden of diseases that require emergency care systems for pre-hospital treatment and/or transport to a hospital for additional treatment is increasing globally, creating a growing need for pre-hospital emergency services. Similar to how traumatic and non-traumatic illnesses like COVID-19 and traffic accidents are driving up the need for critical care in emergency rooms [1,2]. RTAs are thought to be the cause of 1.25 million deaths annually and 50 million injuries worldwide [3], therefore, providing critical pre-hospital care is important for those victims [4,5]. An integrated Emergency Medical Services (EMS) system should be in place in every health system around the globe to handle public health emergencies from the perspective of dimensions of quality of care including health equity, patient-centeredness, and time-centeredness [6].

In low-income nations, national health systems must provide emergency medical services. Low-income countries' governments and ministries of health need to pay close attention to the evolution of EMS in those nations and make sure that any such evolution is both evidence-based and suited to those nations' requirements. More importantly, the context and implementation of EMS should help health equity and not widen existing health disparities [[7], [8], [9]]. Emergency care can make an important contribution to reducing avoidable death and disability in low and middle-income countries [7,10]. In Ethiopia, evidence showed that the complexity and magnitude of injuries in general are increasing. In this regard, the fragmentation of information and evidence seems evident [11].

An emergency condition is defined as one that requires intervention within minutes to hours to reduce the chance of disability and death and improve health outcomes [12,13]. The top emergency conditions and diseases around the world in 2015 were injuries from accidents, falls, and burns (22 %), heart attacks (17 %), lung infections (11 %), and strokes (7 %) [14]. The prevalence of emergency conditions in Ethiopia is also very high and 28 % of emergency room visits are related to trauma [15,16].

The burden of emergency medical conditions is borne mostly by poorer nations, with a 6 % increase in deaths of adults and children due to emergency conditions between 1990 and 2015 [14]. Another study also reported that 54 % or 24.3 million global deaths are amenable to emergency care systems [17]. Ethiopia has one of the highest mortality burdens from emergency conditions. It accounts for 1154 per 100,000 people death and disability-adjusted life years of 47,728 per 100,000 people [18]. In low-middle income (LMIC) nations, no or limited access to EMS results in the development of as many as 45 % preventable deaths and 35 % avoidable disability-adjusted life years [10,19,20]. Due to the low access to the EMS system, the patient's stay in the hospital increased, early discharge from the emergency department decreased, and the death rate increased [21,22].

Factors affecting EMS use were divided into categories that included culture/community, infrastructure, communication/coordination, transport, equipment, and personnel [[23], [24], [25]] According to a systematic review conducted by Koronji et al. lack of transportation was a common problem, with 55 % of articles reporting this as a hindrance to EMS. Ambulances were the most commonly mentioned (71 %), mode of transporting patients. However, many patients still relied on alternative means of transportation such as hired cars, and animal-drawn carts. Sixty-one percent of articles identified a lack of skilled personnel as a key barrier. Forty percent of the systems identified in the review described a uniform access phone number for emergency medical service activation [10].

The limited availability of resources, shortage of facilities and EMS technicians, financial constraints, and infrastructure gaps still hinder the development and implementation of integrated EMS systems. In Ethiopia, the program was in its infancy, there were insufficient partners and stakeholders, and there was a lack of motivation for emergency medical services in Ethiopia. There were also shortages of trained emergency medical providers and an uneven distribution of those who were available [26]. To our knowledge, there is little information in the literature about pre-hospital EMS utilization in the Hawassa city. Therefore, little is known in Ethiopia about aspects of pre-hospital care generally during an emergency state. Thus, the purpose of this study is to evaluate the extent and factors linked to EMS utilization at the community level in Hawassa, Ethiopia. By answering the following questions; what percent of patients utilized emergency medical service? and is there an association between the dependent and independent variables?

## 2 Methods and materials

### 2.1 Study area, period, and design

A facility-based cross-sectional study was conducted from March 15 to April 15, 2023, in Hawassa city. It is located 275 km from Addis Ababa. Administratively, which is divided into eight sub-cities and 32 kebeles, there are 15 government-run health facilities. One Comprehensive Referral Hospital, 1 general hospital, 2 primary hospitals, and five private hospitals (one general and four primary hospitals) were found in the city. According to the Hawassa City Health Department population projection for the 2015 Ethiopian Fiscal Year is 409,811. The city has an estimated number of 20 ambulances transporting patients from the scene to the hospitals, according to the department's emergency medical service unit.

The study was conducted in the emergency unit of four public hospitals namely, Tula Primary Hospital, Motite-Furra Primary Hospital, Adare General Hospital, and Hawassa University Comprehensive Specialized Hospital (HUCSH) which provides emergency and critical care services in Hawassa City. Tula Primary Hospital serves more than 750,000 population. The hospital has 1 ambulance and a total of 54 admission beds. Similarly, Motite-Furra Primary Hospital provides different services for more than 77 thousand population and it has over 20 beds and 5 Ambulances. Adare General Hospital provides a secondary level of health care for the over 1.3 million population, has more than 108 beds in the different units of the hospital and it is providing EMS with two Ambulances. On the other hand, HUCSH is a tertiary-level hospital which comprises of different units. It has over 516 beds and 3 Ambulances which provide interfacility transport and EMS in some instances.

### 2.2 Study population

#### 2.2.1 Source population

The source populations were all clients who visited the emergency units of four public hospitals in Hawassa City during the study period.

#### 2.2.2 Study population

We randomly selected clients who visited the emergency units of four public hospitals in Hawassa City during the study period and fulfilled the selection criteria.

### 2.3 Sample size determination

The sample size was determined in two ways. The first one is using the single population proportion formula by considering the following assumptions: 95 % confidence interval, 5 % margin of error (d), and taking the proportion of EMS utilization 50 % since there is no previous study.

After adding a 10 % non-response rate the final calculated sample size was 422.

The second sample size was calculated using two population proportion formulas by using Epi Info Version 7 software using variables frequently associated with EMS utilization (Table 1).

**Table 1:** Sample size determination using two population proportion formulas from significantly associated factors.

Variables	CI	Exposed to unexposed ratio	Power	% of outcome in un-exposed	OR	Sample size including 10 % NRR
Know free of charge gov't ambulance service (Yes/No) [27]	95	1:1	80	37.67 %	2.8	152
Intention to call if it was a toll-free three-digit number (Yes/No) [27]	95	1:1	80	34.52 %	2.6	176
Prior use of Ambulance (Yes/No) [28]	95	1:1	80	28.44 %	4.1	88

Even though we have calculated the sample size by using both the single population proportion formula and two population proportion formulas by using Epi Info V.7. The larger sample was taken from **the** sample size calculated by **the** single population proportion formula by adding 10 % a non-response rate, the final sample size was 422.

### 2.4 Sampling technique and procedure

**Fig. 1:**
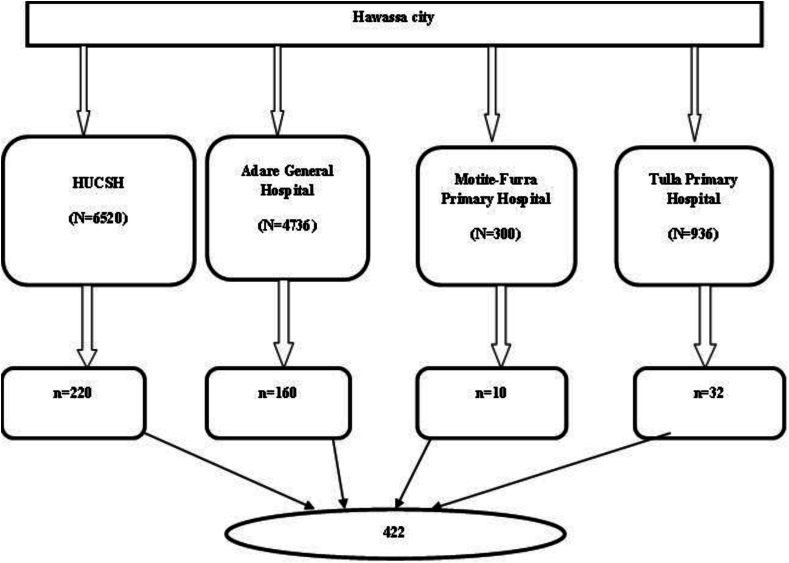
Schematic presentation of sampling technique for utilization and associated factors of EMS in Hawassa city, 2023.

All four public hospitals in Hawassa City (Hawassa University Comprehensive Specialized Hospital, Adare General Hospital, Motite-Furra Primary Hospital, and Tula Primary Hospital) were included in this study to acquire the required number of cases. The allocation of the required number of study subjects to each health facility was determined proportional to population size from reviewing the 2014 Ethiopian Fiscal Year (EFY) patient flow at emergency units from health management information systems (HMIS) records. The proportional-to-size method was employed to select the sample allocated to each selected hospital based on the number of emergency cases in the hospital. A consecutive sampling method was employed to include the sample allocated from each hospital (Fig. 1).

### 2.5 Eligibility criteria

Consecutive emergency patients who arrived directly from the scene and critically ill emergency patients who had a surrogate were included in the study. On the other hand, inter-facility transfers from another healthcare facility and patients linked from the outpatient department to the emergency were excluded from the study.

### 2.6 Variables

Utilization of emergency medical services was the dependent variable. **S**ocio-demographic factors like (age, sex, educational status, occupation, monthly income), patient, health facility & health care related factors (type of case, types of emergencies, availability of ambulance service, ambulance waiting time, emergency medical technician**,** and knowledge and perception (knowledge of EMS, perception about ambulance usage) were the independent variables.

### 2.7 Operational definition

**Emergency medical service**: Give medical assistance before the patients reach to hospital, or care given on scene and medical ambulance during transportation.

**EMS utilization**: If the respondent got any care at the emergency scene, was considered to have utilized EMS.

### 2.8 Data collection tools and procedures

The data were collected using a structured questionnaire prepared by reviewing different literature [15,[29], [30], [31]]. The questionnaire was developed after reviewing relevant literature on the subject to include all the possible variables that address the study's objective. The questionnaire has four sections. The first part includes socio-demographic factors to assess the socio-demographic profile of study participants such as sex, age, marital status, occupation, educational status, monthly income, and area where they are living. The second part includes questionnaires that addressed the clinical profile of study participants with six questions. The third part includes EMS utilization which was assessed by four questions. If the respondent got any care at the emergency scene, was considered to have utilized EMS. The fourth section includes the previous history of EMS utilization which was assessed by structured questionnaires. The last section includes knowledge, perception, and accessibility-related factors assessed by structured yes/no questionnaires. The data was collected by an interview administered questionnaire by four diploma nurses who were employed as data collectors and one health officer was assigned as supervisor. They were selected from the catchment area based on their previous experience in research. During the data collection period, the target study populations were selected based on the sample size of the study which is a total of 422. All the participants who visited those public Hospitals were selected consecutively and were checked for inclusion criteria and then continued interviews until the sample size was reached.

### 2.9 Data quality assurance

The questionnaire was first developed in English then translated into Amharic language and back to English to ensure its consistency. The interview was conducted using an Amharic version of a questionnaire as the Amharic language is spoken by a majority of the residents in Hawassa City. Three days of intensive training for data collectors and supervisors, about the purpose, the tools, and an overall data collection procedure was given. In addition, a pre-test was carried out on 5 % of the total sample size at Yirgalem Hospital, which is closer but outside the proposed study area to assess the clarity, consistency, and understandability of the questionnaires. Face validity was done to assess the questionnaires whether the items of each domain were sensitive, appropriate, and relevant to the study. A question with a higher proportion of non-responses and missing values was modified. The supervisors meet the interviewers, at least once a day to collect the questionnaires and discuss any problems that arise. Every morning the supervisors meet with principal investigators and reports are presented about the results of the previous day. Every week there was a meeting of principal investigators with supervisors and interviewers where progress and any problems that arose during the study were solved. The collected data were reviewed and checked for completeness and relevancy by the supervisors and principal investigators.

### 2.10 Data processing and analysis

The data were collected via the Kobo toolbox and then exported to SPSS version 27 for data analysis. Descriptive statistics were used, using frequency, percentage, mean, and standard deviation. Binary logistic regression models were used and, bivariate and multivariate analysis were performed, and only variables with p-value <0.25 in the bivariate analysis were further entered into the multivariate logistic regression model. An odds ratio with a 95 % confidence level was used to determine the strength of the association. In the multivariate analysis, independent variables with p-value <0.05 were considered significant. Hosmer and Lemeshow goodness of fit test were used to assess the overall fitness of the model and the result was 0.372.

### 2.11 Ethical consideration

The ethical approval letter was obtained from the Research and Ethical Review Board of Hawassa University, College of Medicine and Health Science (Reference number: IRB/296/15). Official letters of permission were taken from the Hawassa city health department to each hospital and permission was secured from the selected hospitals before the beginning of data collection. All participants were fully informed then written consent was obtained from each of the respondents. After the purpose of the study had been explained, respondents were allowed to decide whether or not to participate in the study.

### 2.12 Socio-demographic characteristics of the respondents

A total of 422 participants had completed the interview, making a response rate of 100 %. The mean (±SD) age of the study participants was 33.73 (±14.67). Regarding the sex of the study participants, 55 % of the study respondents were males and 42.2 % of the study respondents were in the age group of 18–27 years while 15.2 % of them were ≥48 years of age. Majorities (58.1 %), of the respondents were single by marital status and 20.9 % of the study respondents were housewives. Regarding the educational status of the respondents, 34.4 % of them had college and above educational level. On the other hand, 58.8 % of the respondents had more than 2000 Birr monthly income and 60.7 % of them were urban residents (Table 2).

**Table 2:** Socio-demographic characteristics of study respondents selected among emergency patients visiting public hospitals of Hawassa city, Sidama Region, Ethiopia, 2023 (n = 422).

Characteristics	Frequency	Percent (%)
**Sex of the respondent**		
Male	232	55.0
Female	190	45.0
**Age of the respondent (Years)**		
18-2	178	42.2
28-37	123	29.1
38-47	57	13.5
≥4	64	15.2
**Marital Status**		
Single	151	35.8
Married	245	58.1
Widowed	18	4.3
Divorced	8	1.9
**Occupation**		
Employed	80	19.0
Unemployed	34	8.1
Housewife	88	20.9
Farmer	70	16.6
Student	91	21.6
Merchant	20	4.7
Other	39	9.2
**Educational status**		
Can't read and write	75	17.8
Primary school	135	32.0
Secondary school	67	15.9
College and above	145	34.4
**Residence**		
Urban	256	60.7
Rural	166	39.3
**Monthly income**		
<1000 ETB	136	32.2
1000–1999 ETB	38	9.0
≥2000 ETB	248	58.8

### 2.13 Clinical profile of the respondents

In the current study, researchers investigated the site of emergency conditions experienced by a group of study participants. The majority of the participants, approximately 62.3 % (263 individuals), reported encountering emergencies at their homes, meanwhile, 136 (32.2 %) reported encountering emergencies on the road. Regarding the type of cases experienced by the participants, it was found that medical cases were the most prevalent. Approximately 52.4 % (221 individuals) of the cases reported by the participants were related to medical emergencies. In addition to medical cases, a significant proportion of participants, 38.2 % (161 individuals), reported encountering psychiatric emergencies. Additionally, from a total of study participants, 148 (35.1 %) of them experienced Injury/Trauma. Furthermore, the researchers identified the most common type of injury among the study participants, which were road traffic accidents (RTAs). This category accounted for approximately 43.2 % (64 injuries) of all reported injuries (Table 3).

**Table 3:** Clinical profile of the study respondents selected among emergency patients visiting public hospitals of Hawassa city, Sidama Region, Ethiopia, 2023 (n = 422).

Characteristics	Frequency	Percent (%)
**Site of emergency condition**		
Home	263	62.3
Roads	136	32.2
Workplace	8	1.9
Other	15	3.6
**Type of cases**		
Pediatrics	13	3.1
Medical	221	52.4
Oby/Gyn	25	5.9
Surgical	2	0.5
Psychiatry	161	38.2
**Nature of your acute illness**		
Labor and delivery-related	25	5.9
Children under 13 years	11	2.6
Acute illness	218	51.7
Injury/Trauma	148	35.1
Other	20	4.7
**Type of Injuries**		
RTA	64	43.2
Fall down	30	20.3
Fighting	24	16.2
Stab wound	15	10.1
Gunshot	6	4.1
Others	9	6.1

Concerning the emergency department triage of the participants, 281(66.6 %) of them had yellow (meaning serious injuries but not immediately life-threatening), 43(10.2 %) of them had green (walking wounded/minor injuries) and 98(23.2 %) of them had red (severe injuries but high potential for survival with treatment; taken to collection point first) triage color.

### 2.14 Previous history of EMS utilization

Regarding ever utilization of an ambulance service for emergency conditions for self or family members, only 133 (31.5 %) of the study respondents have ever used an ambulance service. Regarding the location of emergencies, the home was found to be the most frequent place where emergencies occurred, with 265 (62.8 %) of the respondents reporting this. In terms of ambulance utilization, 39 (29.3 %) used the Motitte Furra Hospital dispatch center (Anekira) ambulance and 15 (11.3 %) used the Red Cross ambulance. When it comes to acquiring information about ambulance services, local media was the most frequently mentioned information channel, with 60 (45.1 %) of the respondents relying on it. Furthermore, family members were also a commonly mentioned source of information, with 24 (18.0 %) of the participants relying on them for ambulance-related information. Overall, this research highlights the respondents' understanding of emergency medical services, the prevalent occurrence of emergencies at home, the utilization of different ambulance providers, and the information channels frequently used by the study participants (Table 4).

**Table 4:** Previous history of EMS utilization of study respondents selected among emergency patients visiting public hospitals of Hawassa city, Sidama Region, Ethiopia, 2023 (n = 422).

Characteristics	Frequency	Percent (%)
**Ever used an Ambulance service**		
Yes	133	31.5
No	289	68.5
**What does an emergency medical service mean?**		
Service that provides emergency medical treatment	301	71.3
Service that provides emergency transportation	107	25.4
Service that provides emergency plumbing repairs	14	3.3
**The place of emergency happened**		
At home	265	62.8
At school	9	2.1
At street	80	19.0
At work	68	16.1
**Type of ambulance used**		
Red Cross	15	11.3
Anekira Ambulance	39	29.3
Private Ambulance	13	9.8
Other	66	49.6
**Information channel you get information about the Ambulance information**		
Mass media	26	19.5
Local media	60	45.1
From my family	24	18.0
From friends	23	17.3

### 2.15 Knowledge, perception, and accessibility of EMS

The research findings reveal insights into the knowledge, perception, and accessibility-related factors influencing the use of EMS. Regarding knowledge, the majority (90.8 %) were aware of the availability of free government ambulance services, and nearly half (48.3 %) knew of free RCA ambulance services. Additionally, the majority (88.9 %) of the study respondents perceived ambulances as safer than taxis, while a smaller minority (31.0 %) believed taxis to be faster. Moreover, an overwhelming majority (86.3 %) considered ambulances to be superior to taxis. Looking at the accommodation of EMS services, a substantial majority (92.2 %) recognized the importance of ambulances for patients' conditions. Concerning perception, over half (50.7 %) of the respondents perceived the presence of an adequate number of ambulances. Positively, a considerable majority (71.8 %) had a favorable perception of the ability to obtain an ambulance on-call. Notably, 84.1 % expressed an intention to call an ambulance if it were toll-free. In terms of accessibility, 18.2 % of respondents reported expected ambulance response times exceeding 60 min during peak traffic hours, while 31.3 % anticipated response times of fewer than 15 min during non-peak hours. Furthermore, a minority (25.6 %) of respondents had ever made an ambulance call, and an even smaller percentage (30.1 %) had utilized ambulance services in the past. Furthermore, respondents attributed various roles to ambulances, with 51.9 % identifying the transportation of ill individuals, 48.6 % recognizing the transportation of trauma victims, 30.1 % acknowledging inter-facility patient transport, and 5.9 % associating ambulances with the transportation of corpses (Table 5).

**Table 5:** Knowledge, perception, and accessibility-related factors of EMS use of study respondents selected among emergency patients visiting public hospitals of Hawassa city, Sidama Region, Ethiopia, 2023 (n = 422).

Characteristics		Frequency	Percent (%)
Availability	**Perception of enough ambulance**		
	Yes	214	50.7
	No	208	49.3
	**Can name a nearby ambulance number**		
	Yes	116	27.5
	No	306	72.5
	**Having the RCA ambulance number**		
	Yes	62	14.7
	No	360	85.3
	**Ever made an ambulance call**		
	Yes	108	25.6
	No	314	74.4
	**Ever used an ambulance service before**		
	Yes	127	30.1
	No	295	69.9
Accessibility	**Expected ambulance response time during peak traffic hours**		
	≤15 min	151	35.8
	16–59 min	194	46.0
	≥60 min	77	18.2
	**Expected ambulance response** **Time during non-peak traffic hours**		
	≤15 min	132	31.3
	16–59 min	202	47.9
	≥60 min	88	20.9
	**Perception of getting ambulance on-call**		
	Yes	303	71.8
	No	119	28.2
Affordability	**Awareness of free gov't ambulance**		
	Yes	342	81
	No	80	19
	**Awareness of free RCA**		
	Yes	204	48.3
	No	218	51.7
	**Intention to call an ambulance if it was toll-free**		
	Yes	355	84.1
	No	67	15.9
Acceptability	**Perception of high-quality care by ambulance technician**		
	Yes	215	50.9
	No	207	49.1
	**The perception that Ambulance is safer than taxi**		
	Yes	375	88.9
	No	47	11.1
	**The perception that taxis are faster than ambulance**		
	Yes	131	31.0
	No	291	69.0
	**The perception that Ambulance is better than a taxi**		
	Yes	364	86.3
	No	58	13.7
Accommodation	**The perception that an ambulance is important for a patient's condition**		
	Yes	389	92.2
	No	33	7.8
	**Importance of ambulance services**		
	Transporting of ill person	219	51.9
	Transporting of trauma victims	205	48.6
	Inter-facility patient transport	127	30.1
	Transporting corpse	25	5.9

### 2.16 Utilization of emergency medical service

This study found that only 24.9 % (95 % CI: 21.1–29.4) of the study participants utilized emergency medical services. Among those who did receive care, the most common type of care was first aid, received by 82.8 % of the participants. It was also found that the majority of care providers were relatives of the participants, accounting for 52.4 % of the cases. Mode of transport to the health facility was also assessed and most 170 (40.3 %) used taxis, 70 (16.6 %) private vehicles, and 15.9 % used Ambulances to come to the hospital. The time of arrival to the first health facility varied, with 40.3 % of participants arriving within 30–60 min. This suggests a potential time delay in accessing care for some individuals. Regarding gaps identified during the EMS process, the most commonly reported issue was time delay, as mentioned by 38.1 % of the study respondents.

The findings also revealed that 27 respondents, accounting for approximately 25.7 % of the total, reported being very satisfied with the EMS they received. However, it was notable that a significant proportion of the participants, specifically 22 individuals or 21 %, expressed their dissatisfaction with the service provided by the EMS team. These results indicate that while a considerable number of study respondents were content with the EMS they received, there is room for improvement. It is crucial to address the concerns raised by the dissatisfied respondents to enhance the quality of service provided by the EMS team. When asked about the reasons for not receiving care, 60.9 % of the respondents cited a lack of knowledge among care providers. Additionally, 4.4 % mentioned a lack of equipment as a factor hindering the provision of care. Overall, these findings highlight the need to improve EMS access and address issues such as time delay, lack of equipment, and gaps in knowledge among care providers (Table 6).

**Table 6:** EMS utilization-related characteristics of emergency patients visiting public hospitals of Hawassa city, Sidama Region, Ethiopia, 2023.

Characteristics	Frequency	Percent (%)
**Received medical help**		
First aid	87	82.8
Positioning	16	15.2
Others	2	1.9
**Care provider**		
Relatives	55	52.4
Bystander	16	15.2
Trained ambulance staff	22	21.0
Police	12	11.4
**Time of arrival to a first health facility**		
Less than 15min	88	20.9
15–30 min	164	38.9
30–60min	170	40.3
**Mode of transport**		
Taxi	170	40.3
Ambulance	67	15.9
Private vehicle	70	16.6
Carried by people	23	5.5
Walking	21	5.0
Bajaj	25	5.9
Motorbike	46	10.9
**Gaps identified while receiving the EMS**		
Time delay	40	38.1
Knowledge and skills gap	25	23.8
Lack of equipment in the Ambulance	32	30.5
Other	8	7.6
**Satisfied by the EMS**		
Very satisfied	27	25.7
Satisfied	34	32.4
Somewhat satisfied	22	21.0
Not satisfied	22	21.0
**The main reasons for not receiving care**		
Lack of knowledge	193	60.9
Lack of equipment	14	4.4
Fear of procedure	7	2.2
Fear of medico-legal issues	12	3.8
Others	91	28.7

### 2.17 Factors associated with EMS utilization

The research findings show that several factors are associated with EMS utilization. After adjusting for confounding variables, it was found that residence (urban), ever utilization of ambulance service, the ability to name the RCA number, and knowledge of the presence of free government ambulance were independent predictors of EMS utilization.

Urban residents were 3.48 times more likely to receive any type of care at the emergency scene compared to their rural counterparts (AOR = 3.48, 95%CI: 1.69–7.16). Participants who had ever used ambulance service were 2.37 times more likely to receive EMS compared to those who had never used ambulance service (AOR = 2.37, 95%CI: 1.21–4.67). In addition, participants who had awareness of the presence of free government ambulance had 3.74 times increased odds of receiving EMS compared to those who were unaware (AOR = 3.74, 95%CI: 1.46–9.59). Furthermore, participants who had Red Cross Association ambulance numbers were 2.64 times more likely to receive care at the emergency scene compared to those who could not (AOR = 2.64, 95%CI: 1.20–5.83) (Table 7).

**Table 7:** Results of bi-variable and multi-variable analysis of study respondents selected among emergency patients visiting public hospitals of Hawassa city, Sidama Region, Ethiopia, 2023 (n = 422).

Characteristics	Received any form of care at the scene		COR (95%CI)	AOR (95 % CI)
	Yes	No		
**Age of the respondent**				
18–27 years	49	129	1	1
28–37 years	35	88	1.05 (0.63,1.75)	0.94(0.49,1.80)
≥38 years	21	100	0.55 (0.31,0.98)	0.78(0.39,1.59)
**Monthly income**				
<1000 ETB	25	111	1	1
1000–1999 ETB	11	27	1.81(0.79,4.13)	1.61(0.61,4.31)
≥2000 ETB	69	179	1.71(1.02,2.87)	1.32(0.67,2.59)
**Residence**				
Urban	89	167	4.99(2.81,8.89)	3.48(1.69,7.16) *
Rural	16	150	1	1
**Educational status**				
Below secondary school	32	178	1	1
Secondary school	21	46	2.54(1.34,4.81)	1.52(0.66,3.46)
College and above	52	93	3.11(1.87,5.16)	1.45(0.74,2.85)
**Mode of transport**				
Taxi	37	204	0.58(0.20,1.68)	0.71(0.23,2.23)
Ambulance	38	29	4.19(1.38,12.78)	2.15(0.61,7.51)
Private vehicle	20	50	1.28(0.41,3.96)	1.06(0.31,3.59)
Carried by people	5	18	0.89(0.22,3.64)	1.12(0.23,5.02)
Walking	5	16	1	1
**Ever used an ambulance service**				
Yes	60	73	4.46(2.79,7.11)	2.37(1.21,4.67) *
	45	244	1	1
**Can name a nearby ambulance number**				
Yes	53	63	4.11(2.56,6.59)	1.16(0.55,2.41)
No	52	254	1	1
**Have the RCA ambulance number**				
Yes	36	26	5.84(3.31,10.31)	2.64(1.20,5.83) *
No	69	291	1	1
**Perception of enough ambulance**				
Yes	64	150	1.74(1.11,2.73)	1.16(0.64,2.09)
No	41	167	1	1
**Awareness of free gov't ambulance**				
Yes	99	243	5.03(2.12,11.92)	3.74(1.46,9.59) *
No	6	74	1	
**Perception of high-quality care by ambulance technician**				
Yes	68	147	2.13(1.35,3.36)	1.52(0.87,2.66)
No	37	170	1	

## 3 Discussion

This study aimed to assess the utilization of EMS and its associated factors among patients visiting public hospitals in Hawassa City, Sidama Region, Ethiopia. In this study, there were 105 (24.9 %) of the participants who had received care by EMS.

This finding is comparable to studies conducted in different parts of the world. For example, a study in Mekelle City, Ethiopia reported a utilization rate of EMS of 22.7 % [27]. However, the utilization rate in this study was higher than a study conducted in Addis Ababa, Ethiopia, which reported a rate of 20.3 % [16]. In the United States, a study assessing EMS utilization among children aged <19 years found that pediatric patients represented 13 % of all EMS transports [32]. In Jakarta, Indonesia, a study found that the majority of trauma patients traveled to the hospital using a motorcycle or car, with only 9.3 % using an ambulance [33]. The discrepancy in findings could be due to differences in study settings and populations.

Additionally, the utilization rates in this study were lower than another study conducted in Addis Ababa Ethiopia, which reported a rate of 46.2 % for pre-hospital care [11], and another study in Addis Ababa, Ethiopia, which reported a utilization rate of 59.2 % [34]. Similarly, a study in Nairobi County, Kenya reported a utilization rate of 37 % for ambulance services [35]. This difference could be attributed to the fact that higher utilization rates were observed in capital cities with better access to healthcare services.

Residing in the urban areas had higher odds of receiving any type of care at the emergency scene compared to their rural counterparts. This finding is supported by a study conducted in Bavaria, Germany, which found higher emergency rates with pre-hospital physicians in urban municipalities [36]. It is also consistent with a study conducted in Saudi Arabia [6]. The reason might be due to urban residents having better access to media and information about ambulance services which might have an impact on the awareness of the public which in turn might make it easy to access and utilize the service [37,38].

Additionally, participants who had ever utilized ambulance services were 2.37 times more likely to receive EMS compared to those who had never used ambulance services. This result is in line with other studies, such as one conducted in Jimma City, Ethiopia, which found a positive association between prior ambulance use and ambulance utilization [28]. This result might be because familiarity with the service makes it easy to access and utilize the EMS. This indicates that the satisfaction of patients in, the EMS is important and might affect future utilization.

Furthermore, participants who had awareness of the presence of free government ambulance services had 3.74 times increased odds of receiving EMS compared to those who were unaware. This finding is consistent with a study that reported participants with knowledge of free-of-charge government ambulance services were 2.8 times more likely to call for an ambulance [27]. Another study conducted to assess utilization of ambulance service during labor and delivery also indicated that mothers who were aware of free ambulance service were 3 times at increased odds of utilizing the service. The justification could be, that knowing the availability of free-of-charge ambulance service might have increased the level of confidence which assisted in better utilizing the service [27,39]. This study also found that participants who have Red Cross ambulance phone numbers were more than 2.6 times more likely to receive EMS. This finding is supported by a study conducted in Ethiopia, which found that participants who knew the Red Cross ambulance phone number were 2.6 times more likely to call for ambulance services in case of an emergency [27]. As the urgency of perceived emergency conditions leaves people highly vulnerable to financial pressures, knowing the availability of EMS like ambulance services provided by RCA might improve care care-seeking behavior of patients and their families. This might in turn improve the utilization of EMS [27,39].

Overall, this study provides valuable insights into EMS utilization and its associated factors in Hawassa City, Sidama Region, Ethiopia. The findings highlight the importance of improving access to healthcare services, increasing awareness of available ambulance services, and enhancing emergency response systems in both urban and rural areas.

### 3.1 Strengths and limitations of the study

#### 3.1.1 Strengths of the study

Even though there were many studies conducted to assess utilization determinants of EMS use in Ethiopia, no study was conducted to assess EMS utilization status and the associated factors in the study area so it can be used as a baseline entry point for other studies. Furthermore, the study covered all of the public hospitals in Hawassa city.

#### 3.1.2 Limitations of the study

The interpretation of this result should consider the following limitations. First, the largest limitation is selection bias, since this is a hospital-based study; there is a chance that participants in this setting might be a high-risk group, so this could have led to overestimation. Second, as the cross-sectional study design was used, the study could be limited by a lack of temporal relationship. Third, it is not free of recall bias.

## 4 Conclusion

The findings of the current study highlight several important aspects of EMS in Hawassa City. The study reveals that the utilization of emergency medical services in the study area is alarmingly low, and a significant proportion of individuals did not receive any medical help at the emergency scene, indicating a potential gap in emergency response services.

Several factors including residence, prior usage of ambulance services, awareness of the availability of free government ambulance services, and knowledge about the necessary RCA numbers were found to significantly impact the utilization of emergency medical services. Overall, this research underscores the need for comprehensive improvements in EMS in Hawassa City. By addressing the gaps identified in this study, the city can enhance its emergency response capabilities, reduce morbidity and mortality rates, and ultimately improve the overall health outcomes of its residents. This highlights the need for targeted interventions to increase awareness, accessibility, and utilization of emergency medical services in the study area. It is essential to enhance communication systems, establish a centralized emergency response center, and implement efficient dispatch systems. Addressing the knowledge and skills gap requires investing in regular and standardized training programs for EMS providers. This training should be tailored to local needs and implemented in collaboration with medical institutions and relevant stakeholders. The curriculum should focus on enhancing emergency assessment, first aid, critical care procedures, and the proper usage of medical equipment. Regular inventory checks, maintenance schedules, and the provision of necessary life-saving devices and medication must be prioritized. Additionally, a well-structured system for resource allocation and regular monitoring of equipment status is necessary.

## 5 Recommendations

Based on the findings of the study, the following recommendations are forwarded:

Developing a centralized emergency response center, implementing effective dispatch systems, and improving communication networks are all critical steps in closing the time delay gap. Response times can be shortened and incident locations can be quickly identified by enhancing cooperation among emergency service providers and incorporating GPS technology.

EMS providers need to invest in frequent, standardized training programs to close the knowledge and skills gap. These programs should be comprehensive. In cooperation with relevant stakeholders and medical institutions, this training should be customized to the requirements of the community. Strengthening emergency assessment, critical care procedures, first aid, and the appropriate use of medical equipment should be the curriculum's primary objectives.

Equipment acquisition and maintenance close the equipment gap in ambulances, and a concentrated effort should be made to guarantee that ambulances are fully stocked with necessary medicines. Prioritizing tasks such as routine inventory checks, maintenance schedules, and the provision of essential life-saving equipment and medication is imperative. Furthermore, a systematic approach to resource distribution and routine equipment status monitoring is required.

## Funding statement

There is no special funding for this study.

## Consent for publication

Not applicable.

## Data availability statements

The data will be available upon request by the correspondence author.

## CRediT authorship contribution statement

**Zelalem Mekonen:** Writing – original draft, Investigation, Formal analysis, Data curation. **Wegene Jemebere:** Writing – review & editing, Writing – original draft, Supervision. **Aklile Tsega Chekol:** Writing – review & editing, Writing – original draft. **Fikru Tadesse:** Writing – review & editing, Writing – original draft. **Yacob Abraham Borie:** Writing – review & editing, Writing – original draft. **Ezedin Mola:** Writing – review & editing, Writing – original draft. **Mastewal Aschale Wale:** Writing – review & editing, Writing – original draft. **Yunuka Marufa Tunushe:** Writing – review & editing, Writing – original draft. **Yared Reta:** Writing – review & editing, Writing – original draft. **Amdehiwot Aynalem:** Writing – review & editing, Writing – original draft. **Beyene Feleke:** Writing – review & editing. **Gelane Geleto Gobena:** Writing – review & editing. **Bereket Beyene:** Writing – review & editing, Writing – original draft. **Tomas Yeheyis:** Writing – review & editing, Writing – original draft, Supervision.

## Declaration of competing interest

The authors declare that they have no known competing financial interests or personal relationships that could have appeared to influence the work reported in this paper.
